# Tau and mTOR: The Hotspots for Multifarious Diseases in Alzheimer's Development

**DOI:** 10.3389/fnins.2018.01017

**Published:** 2019-01-10

**Authors:** Zeba Mueed, Pallavi Tandon, Sanjeev Kumar Maurya, Ravi Deval, Mohammad A. Kamal, Nitesh Kumar Poddar

**Affiliations:** ^1^Department of Biotechnology, Invertis University, Bareilly, India; ^2^King Fahad Medical Research Center, King Abdulaziz University, Jeddah, Saudi Arabia; ^3^Enzymoics, Hebersham, NSW, Australia; ^4^Novel Global Community Educational Foundation, Hebersham, NSW, Australia

**Keywords:** Alzheimer's disease (AD), tau, mTOR, Aβ plaques, Aβ modulators, hyperphosphorylation, NFT's

## Abstract

The hyperphosphorylation of tau protein and the overexpression of mTOR are considered to be the driving force behind Aβ plaques and Neurofibrillay Tangles (NFT's), hallmarks of Alzheimer's disease (AD). It is now evident that miscellaneous diseases such as Diabetes, Autoimmune diseases, Cancer, etc. are correlated with AD. Therefore, we reviewed the literature on the causes of AD and investigated the association of tau and mTOR with other diseases. We have discussed the role of insulin deficiency in diabetes, activated microglial cells, and dysfunction of blood-brain barrier (BBB) in Autoimmune diseases, Presenilin 1 in skin cancer, increased reactive species in mitochondrial dysfunction and deregulated Cyclins/CDKs in promoting AD pathogenesis. We have also discussed the possible therapeutics for AD such as GSK3 inactivation therapy, Rechaperoning therapy, Immunotherapy, Hormonal therapy, Metal chelators, Cell cycle therapy, γ-secretase modulators, and Cholinesterase and BACE 1-inhibitors which are thought to serve a major role in combating pathological changes coupled with AD. Recent research about the relationship between mTOR and aging and hepatic Aβ degradation offers possible targets to effectively target AD. Future prospects of AD aims at developing novel drugs and modulators that can potentially improve cell to cell signaling, prevent Aβ plaques formation, promote better release of neurotransmitters and prevent hyperphosphorylation of tau.

## Introduction

The most prevalent type of dementia, which is known to be occuring worldwide is Alzheimer's disease (AD), affecting more than 40 million people worldwide (Selkoe and Hardy, 2016) and which is expected to get tripled by 2050 (Galvan and Hart, 2016). AD is known to be a prime reason behind 70% of the dementia (Kametani and Hasegawa, 2018). Reports have suggested that almost 2.7% of India's population is wretched by this disease (Stern, 2012). AD is primarily characterized by memory and language impairment, decision making, attention, and orientation (Folch et al., 2018). Apart from AD, other types of dementia include Parkinson's disease (PD), Huntington's disease (HD), Down syndrome etc. (Korolev, 2014). Numerous studies have depicted that aging is one of the major risk factor for AD progression, however the underlined mechanism is still unknown. One of the research studies has given an indication of the fact that vascular mTOR signaling mechanisms can be related to aging and AD (Borlikova et al., 2013). We are aware of the fact that AD is caused due to the failure of nerve cells, but how does it exactly happen is still uncertain. However, certain elements of danger have been identified that greatly enhance the likelihood of AD spread (de Paula et al., 2009).

Several theories have been put forward with regards to the development of AD among which Amyloid and Tau hypothesis are the most prevalent ones which function to promote synaptic and neuronal damage (Borlikova et al., 2013). The amyloid hypothesis states that AD pathogenesis is triggered by the production and aggregation of amyloid-β. The amyloid-β is generated in response to the catalytic cleavage of APP by β and γ secretase. Any disrupment in the normal functioning of β and γ secretase results in the accumulation of Aβ, hindering normal brain functioning. Tau hypothesis in turn states that the hyperphosphorylation of MAP associated protein, tau, promotes the detachment of tau from microtubules resulting in microtubule instability, and the formation of NFT's, a major hallmark of AD (Folch et al., 2018). mTOR, an essential regulator of various cellular, and metabolic processes, plays a critical role in the development of AD. Activation of mTOR inhibits autophagy which promotes Aβ aggregation (Sahab Uddin et al., 2018). It is evident that any deficit in the autophagy-lysosomal system is seen to be associated with the deposition of Aβ in brains; however, a recent research has indicated that the liver metabolism of Aβ may also prove to be a contributing factor in AD pathogenesis (Maarouf et al., 2018). Apart from amyloidogenesis and taupathy, several other reasons may be attributed to AD such as mitochondrial dysfunction, neuroinflammation, cell cycle deregulation etc. Several therapeutic approaches have been discussed to combat AD progression which is known to cut down AD progression at every possible level such as immunotherapy, metal chelators, γ secretase inhibitors, hormonal therapy etc. (Geylis et al., 2005; Kozlowski et al., 2009). However, no single therapy has been successful in complete obstruction of AD progression. Considering the recent strategies which are aimed at developing therapies to effectively target AD, global leaders have established an aim to treat AD completely by 2025 (Cummings et al., 2016).

This review summarizes the role of Aβ and tau and their close interplay with mTOR and autophagy in AD progression as well as their role as a trigger for clinical diseases in promoting AD such as autoimmunity, mitochondrial dysfunction etc. (Lim et al., 2007; Fernandez et al., 2009) along with different therapeutic approaches aimed at targeting AD from progressing.

## Mammalian Target of Rapamycin, mTOR

**Figure 1:**
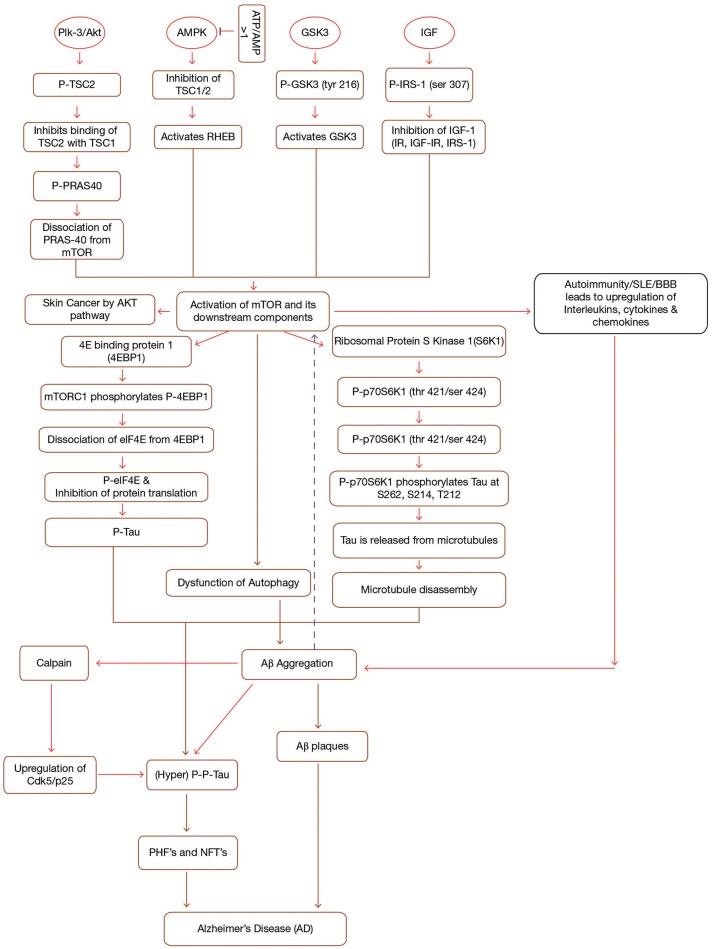
Interplay between mTOR, Aβ and Tau in Alzheimer's disease. mTOR is activated by the activation of a number of upstream components (PI3-K/Akt, GSK3, AMPK, IGF-1) in a sequence specific pathway. Activation of mTOR results in the activation of downstream components (4EBP1 and p70S6K1). Both 4EBP1 and p70S6K1 initiate their cascades ultimately causing hyperphosphorylation of tau, leading to the formation of PHF's and NFT's. mTOR leads to the accumulation of Aβ plaques by inhibiting autophagy. Accumulated Aβ further induces tau phosphorylation and mTOR activation. Additionally, Immune dysfunction, blood-brain barier dysfunction, upregulated Cdk5, and interlukins also contribute to AD. Formations of NFT's and Aβ plaques give rise to AD symptoms. IGF, Insulin Growth Factor; TSC1/TSC2, Tuberous Sclerosis Complex1/2; IRS, Insulin Receptor Substrate; IR, Insulin Resistance; p70S6K1, p70 ribosomal S6 Kinase1; PHF's, Paired Helical Filaments; NFT's, Neurofibrillary Tangles, p, phosphorylation; p-p, hyperphosphorylation; PI3-K, phosphoinositide 3-kinase; AMPK, AMP-activated protein kinase; GSK3, Glycogen Synthase Kinase.

mTOR, which is made up of two complexes (mTORC1 and mTORC2), is a protein kinase (289 kD), known to regulate a number of cellular processes such as proliferation, transcription, translation etc. (Shafei et al., 2017). It has been observed that mTOR activation and signaling in itself is controlled by a variety of upstream and downstream components and an abnormality in this is found to be associated with AD mainly by forming Aβ plaques and neurofibrillary tangles (NFT's) (Cleveland-Donovan et al., 2010). Some important upstream components which activate mTOR are phosphoinositide 3-kinase (PI3-K)/protein kinase B (Akt), glycogen synthase kinase 3 (GSK3), AMP-activated protein kinase (AMPK), and insulin/insulin-like growth factor 1 (IGF-1) (Figure 1). On account of this fact, current research study is mainly focusing on to create inhibitors of mTOR which will eventually help in limiting the progression of AD.

## mTOR Signaling Pathways: Role of Upstream Components in mTOR Activation

### GSK3/PI3-K/Akt and mTOR

GSK3, along with its two isoforms GSK3α and GSK3β, serves as a prime factor in variegated metabolic processes, for instance cell cycle regulation, glycogen metabolism, transcription, translation etc. (Buller et al., 2008). In addition, GSK3 hyperphosphorylates tau at serine and threonine residues and it is also involved in the production of Aβ and other pathological changes coupled with AD (Cai et al., 2015). Hence, GSK3 hyperactivation is considered to be responsible for AD as it acts as a connecting link between amyloid pathology and tau hyperphosphorylation (Figure 1). Research content has given an indication of the fact that GSK3 is also connected to PI3K/Akt/mTOR signaling cascades. So, it can be inferred that both PI3-K/Akt and GSK3 are key signaling cascades which regulate the production of Aβ (Kitagishi et al., 2012) (Figure 1). Recently, it has been discovered that L803-mts, which is an antagonist of GSK3 lessens Aβ formation and re-establishes autophagy by restoration of impaired mTOR signaling pathway (Cai et al., 2012).

Stretton et al. (2015) have pointed out that suppressing GSK3 through inhibitors or gene silencing promotes the phosphorylation of raptor, an essential component of mTOR. This phosphorylation in turn inhibits raptor from interacting with mTOR. The reduced mTOR activity inhibits the phosphorylation of its downstream component which in a way promotes autophagy and thus abstaining AD progression (Stretton et al., 2015).

Research studies suggest that Akt, a serine/threonine kinase (Protein Kinase B, PKB) is activated by class 1-phosphoinositide-3 kinase (PI3K)-phosphoinositide-dependent protein kinase-1 (PDK1) in response to growth factors. The activated Akt is further phosphorylated by mTOR complex II and finally the fully induced Akt activates the mTOR complex I through phosphorylation of tuberous sclerosis complex 2 (TSC2) and PRAS40. Activation of mTOR than leads to the pathological changes coupled to AD. Another important point to note is that PI3-K/Akt acts on mTOR to promote autophagy and in turn enhances Aβ clearance and reduces the hyperphosphorylation of tau (Chong et al., 2012). Thus, it can be concluded that PI3-K/Akt/mTOR signaling has a crucial effect on AD pathology (Figure 1).

#### Insulin/IGF-1/mTOR

It has been indicated that people suffering from diabetes exhibit enhanced mTOR activation which in turn is known to hyperphosphorylate tau. Scientific data portrays that deregulation of Insulin/insulin-like growth factor 1 (IGF-1) signaling leads to neurodegenerative disorders such as AD (Salminen and Kaarniranta, 2010) and several other diseases like cancer (Heni et al., 2012), diabetes, (Fowlkes et al., 2011), etc. Current hypotheses and theories implicate that insulin resistance (IR) is one of the prime cause for the severity of AD.

Insulin resistance (type II diabetes) is accused to be the main cause behind AD development in diabetic people (Boura-Halfon and Zick, 2009). Brandt and Leschik (2004) have suggested that AD pathology may also be limited by specific dietary uptake to improve neurodegenerative defects. It has been observed that diabetic population suffering from AD experiences an up-regulation in phosphorIRS-1 and a down regulation in total IRS-1. Thus, researchers believe that hypoglycemic drugs may be valuable for treating AD (Baker et al., 2011) (Figure 1).

### AMPK and mTOR

AMPK (Adenosine Monophosphate-activated Protein Kinase), a heterotrimeric protein, maintains cell homeostasis (Daval et al., 2006). Both the catalytic as well as regulatory subunits play a crucial role in AMPK activation. Firstly, AMPK is phosphorylated at Thr-172, which exposes the active site on catalytic subunit due to modification in one of the regulatory subunits (c-subunit) (Hardie et al., 1999). In addition to hormones and cytokines, high ATP/AMP ratio has been seen to inactivate AMPK (Kemp et al., 2003). To achieve this, AMPK inhibits all the synthetic pathways and stimulates different catabolic pathways such as β-oxidation and many others (Hardy, 2006). This inactivation of AMPK in turn leads to the inactivation of Tuberous Sclerosis Complex 1 and 2 (TSC1/TSC2). Inactivation of TSC complex activates a small GTPase, Ras homolog enriched in brain (RHEB), known for activating mTOR (Lacher et al., 2011) (Figure 1). It has been seen that AMPK activity decreases with age leading to mitochondrial dysfunction and thereby causing pathogenesis of AD (Priebe et al., 2011).

Research data has indicated that AMPK exaggerates the severity of AD by up-regulation of Aβ species (Kwon et al., 2010) (Figure 1). In addition, AMPK is also involved in cholesterol and sphingolipid metabolism thereby controlling the lipid rafts in the bilayer-membrane system. Thus these lipid rafts are endowed with the ability to form Aβ plaques, in a way inducing AD, mainly by transforming APP (Amyloid precursor protein) processing (Holland et al., 2011).

### mTOR and Aβ

Protein misfolding is one of the main culprits behind build-up of Aβ in AD brains. Aβ is a 36–43 amino acid long protein which is generated when APP is cleaved by two enzymes, β-secretase (β-site APP cleavage enzyme, BACE), and γ-secretase complex (Luo et al., 2016). β-secretase cleaves APP between the Met671 and Asp672 residues producing a soluble extracellular fragment (sAPPb) and a cell membrane-bound fragment (C99) whereas γ-secretase cleaves at either Val711 or Ile713, freeing intracellular domain along with the production of Aβ (Cole and Vassar, 2008). The hydrophobicity of Aβ enables it to self aggregate in multiple forms ranging from small oligomers to fibrils and eventually leading to amyloid plaques in AD. These Aβ deposits inhibit synaptic dysfunction by binding to synaptic receptors (Nisbet et al., 2015). It can be conferred that inhibitors of β/γ-secretase can work against AD in a potent manner. Won et al. (2010) showed that prominent levels of Aβ were observed in AMPK knocked out mice. However, the Aβ production was greatly reduced when the AMPK activator, 5-aminoimidazole-4-carboxamide-1-d-ribofuranoside (AICAR) was injected into the mouse. Therefore, it can be concluded that a control check for both AMPK and APP can help in obstructing Aβ from toxicity. Compelling evidence indicate that Aβ deposits hyperactivate mTOR which in turn hyperphophorylates tau, forming PHF's (Paired helical filaments), and NFT's. Consistent with this idea, cell culture and animal model study indicated that exposing Aβ oligomers by injecting into animal models displays mTOR hyperactivity, however this activity is found to be dose dependent (Oddo, 2012).

### mTOR and Tau

Tau is an important highly soluble microtubule associated protein which helps in the stabilization of microtubule polymer, enhancing microtubule assembly by binding to a hydrophobic residue of tubulin heterodimers (Zhang et al., 2009). Compelling evidences have suggested that activation of mTOR signaling cascade enhances tau pathology since mTOR activation leads to dysfunction of autophagy. Dysfunction of autophagy results in the piling up of Aβ which in turn stimulates tau hyperphosphorylation and thus leading to the development of PHF's and NFT's. On the other hand, inhibition of mTOR activation inhibits taupathy (Tramutola et al., 2017). Studies suggest that hyperphosphorylation of tau at serine/threonine residues, as well as other modifications to this protein disrupt the interactions between tau and microtubule complex and this causes the detachment of tau from microtubule, affecting microtubule stability, and assembly, ultimately forming NFT's and hence, leads to several disorders commonly known as taupathies (de Paula et al., 2009; Cai et al., 2015). Tau kinases as well as tau phosphatases stringently control phosphorylation of tau (Canudas et al., 2005). Moreover, various other kinases such as Cyclin-dependent protein kinase 5 (Cdk5), cAMP-dependent protein kinase (Jicha et al., 1999), stress-activated protein kinases (SAPK) (Buée-Scherrer and Goedert, 2002) rigorously administer the same. Similarly, AMPK also phosphorylates tau at Ser262/Ser356, Ser396, and Thr-231 (Salminen et al., 2011). Upraised level of phosphorylated tau in brain is considered as the biomarker for AD patients (Fagan et al., 2011). Apart from AMPK, tau is phosphorylated by different types of kinases, including GSK3 (Flaherty et al., 2000), protein kinase A (Sengupta et al., 2006), and mitogen-activated protein kinases (Pelech, 1995). On the basis of various research studies, it can be stated that AMPK activation antecedes taupathy. Hyperphosphorylation of tau can be considered as an initiator for PHF's which ultimately aggregate into NFT's (Chen L. et al., 2012) (Figure 1). However, this hyperphosphorylated tau is found to be attenuated by Rapamycin, a potential inhibitor of mTOR which helps in clearing this hyperphosphorylated tau by inducing autophagy of the formed Aβ plaques, PHF's, and NFT's (Spilman et al., 2010).

mTOR acts as an essential regulator of catabolic as well as anabolic processes in response to metabolic state of the cell, playing a crucial role in aging, however the mechanism involved is still unclear. Recent research has portrayed that mTOR attenuation increases the life span of mouse models with the evidence of accumulation of tau (Wilkinson, 2012). Overwhelming data suggests that molecular changes occurring in response to aging may promote taupathy (Bertram and Tanzi, 2005). Thus, deciphering the mechanism of age dependent changes in signaling pathway which promote tau accumulation may provide a possible target for combating AD progression.

### mTOR and Autophagy

Autophagy is a natural cellular defense process to get rid of abnormally aggregated proteins and damaged or degraded organelles. Impairment of the neuronal autophagy-lysosomal system is known to be associated with various AD symptoms. Dysfunction of neuronal autophagy-lysosomal system may lead to ineffective clearance of proteins eventually resulting in neural cell death (Shacka et al., 2008). Consistent with this notion, it has also been postulated, that mTOR modulates APP processing as well as integrates with different signaling pathways such as PI3-K/Akt, GSK-3, AMPK, and Insulin/IGF-1, thus regulating both Aβ production and its clearance (Damjanac et al., 2008). Therefore, modulating the autophagy-lysosomal protein degradation pathway can be an attractive approach for treating AD (Jegga et al., 2011). Autophagy is necessary for clearing misfolded and abnormally aggregated proteins. Thus, a disruption in autophagic process in the form of immature autophagolysosome leading to the formation of authophagic vacuoles (AVs) serves an attractive site for Aβ deposits and amyloid plaques in AD. However, rapamycin, an inhibitor of mTOR lessens AVs from accumulating in a way preventing Aβ aggregation (Yu et al., 2005).

Thus, stringent control of autophagy process can help in treating AD. Furthermore, studies also indicate that mTOR inhibits autophagy which accelerates Aβ production and accumulation of tau (Caccamo et al., 2010) and thus paving a way for AD pathogenesis. Recently, Maarouf et al. (2018), has demonstrated that liver metabolism may also be a contributing factor in Aβ deposition in the neuronal cells. They conducted an experiment using synthetic fluorescein-labeled Aβ40 and Aβ42 spiked into human liver homogenates and found out that AD patients had impaired hepatic Aβ degradation. However, a further research is needed in this respect to clearly specify the role of liver in Aβ degradation.

## Cross Talk Between Aβ and Tau and Their Interplay With mTOR and Autophagy

Scientific community has commonly proposed that amyloid plaques and NFT's are the major hallmarks of AD. Amyloid plaques are the fibrillar aggregates of protein Aβ whereas NFT's are the result of hyperphophorylated tau. Both amyloid and tau pathology are known to be neurotoxic. However, which pathology appears first is still a question of debate. There is increasing evidence that Aβ induces tau pathology. In contrast to this, it has been observed that reduction of tau levels in APP transgenic mice effectively reverses the AD symptoms without even changing the Aβ levels (Roberson et al., 2007). Triple transgenic mouse model which was the combination of both Aβ and tau pathology has clearly documented that both Aβ and tau act synergistically in AD progression (Rhein et al., 2009). Thus, an immunotherapeutic approach to treat AD is now focusing on to develop both anti-Aβ and anti-tau antibodies to effectively encounter AD progression.

mTOR, a culprit of Alzheimer, plays a very crucial in both Aβ and tau pathology. Research study has highlighted that activation of mTOR induces Aβ production and aggregation by direct inhibing autophagy/lysosome system. On the other hand, numerous *in vitro* studies have predicted that mTOR activity is upregulated when cells are induced with Aβ by activating the PI3/Akt pathway. It was observed that Aβ induces hyperphophorylation of both Akt and mTOR. Consistent with this, it has been reported that exposure to Aβ, enhanced the levels of p70S6K and p4E-BP1 which further confirms that Aβ activates mTOR (Bhaskar et al., 2009) (Figure 1). An experiment study has indicated that rapamycin, an mTOR inhibitor, reduces Aβ, and tau pathology in the brains of 3xTg-AD mice. It was also observed that tau pathology in mice was directly linked with Aβ deposits. Thus, it is still unclear that whether rapamycin reduces tau pathology due to changes in amyloid-β or because of direct relation between mTOR and tau. Thus interpreting this relation will not only help in understanding role of mTOR in AD but also in other tau related abnormalities (Oddo et al., 2003).

### Downstream Components of mTOR

The activation of mTORC1 by different growth factors and other environmental stresses leads to the activation of its downstream components, 4E Binding protein 1 (4EBP1), and Ribosomal Protein S Kinase 1 (S6K1). Activation of mTORC1 phosphorylates 4EBP1 and this phosphorylation causes elF4E to dissociate from 4EBP1, which in turn leads to the inhibition of Elf4E dependent translation initiation. Thus the phosphorylated 4EBP1 together with eIF4E contributes to tau toxicity (Li et al., 2005) (Figure 1).

Other downstream component of mTOR signaling is p70 Ribosomal S6 Kinase 1 (P70S6K1). P70S6K1 is phosphorylated at (thr421/ser424) by activated mTORC1, (Pei et al., 2008a) which in turn phosphorylates tau at S262, S214, and T212 (Pei et al., 2008b). Phosphorylation of tau causes its release from microtubules initiating microtubule disruption. Thus, research studies have proposed that p70S6K1 is associated with taupathy and other pathological changes associated with AD (Figure 1) (Pei et al., 2006).

## mTOR and Tau: a Trigger for Clinical Diseases in Promoting AD

### Autoimmunity/Inflammation and AD

Autoimmune diseases are invoked due to loss of self-tolerance because of which they manifest an inflammatory response to self-cells and organs of the body, such as in Multiple sclerosis, Rheumatoid arthritis, Insulin-dependent diabetes mellitus (IDDM) etc. The innate immune response is the primary response against the invasion of pathogens on host. This response integrates with a set of pattern recognition receptors (PRPs) through which local CNS (central nervous system) cells may be triggered to develop innate responses (Amor et al., 2014).

In case of autoimmunity, the mononuclear phagocytes from blood are assembled via brain chemokines, permitting the entry of cells from the bloodstream through the BBB. At early stage, there is loss of BBB homeostasis, leading to the production of proinflammatory cytokines, and suppression of the cerebral blood flow by endothelial cells, which inflame synapse eradication, pilling up, and triggering of microglia. This chronic neuroinflammatory environment increases the high levels of cytokines such as such as IL-1 and IL-6, TNF-α and transforming growth factor-β which promotes the processing of APP into Aβ plaques (da Fonseca et al., 2014). Although microglia and astrocyte cells can boost the clearance of Aβ, but, if they are unable to do that, than there is a build-up of Aβ deposits which induces neuronal death. Microglial inflammatory response such as phagocytosis, an aspect of innate immunity, may remove Aβ from the neuron so that microglia can be neuroprotective by phagocytosing amyloid-β, but on the other hand, microglia activation, and secretion of neurotoxins may also cause neuronal disease. Studies indicate that accumulation of amyloid-β could result in microglia activation which in turn degrades amyloid-β but along with this, the secretion of chemokines, and proinflamatory cytokines increases, which further seems to increase amyloid-β in AD brain (McGeer et al., 1990). Thus, the innate immune system is thought to be biphasic as it is shown that at low Aβ concentration, CD14, and the Toll-like receptors (TLRs) act as neuroprotectant by inducing phagocytosis of Aβ, conversely high Aβ concentrations results in the build-up of neurotoxins which mutilate the nearby neurons (Udan et al., 2008).

### mTOR, a Biomarker for SLE, Autoimmune Disease

It has been stated that in SLE, mTORC1 remains activated whereas mTORC2 activity is reduced (Fernandez et al., 2009). This activated mTORC1 leads to death of CD4-, CD8-, Double negative (DN) T cells in patients suffering from SLE. mTORC1 also results in the increased production of IL-4 which further stimulates B cells to produce anti-DNA antibodies. Similarly, increased activity of mTORC1 has also been reported in patient's suffering from multiple sclerosis. The inhibitor of mTOR obstructs the activation of T cells as well as mTORC1 and also lowers the production of IL-4 in SLE patients which has been seen to be very effective to treat SLE both in animal models as well as in patients. Thus, mTOR has been widely attributed to act as a biomarker for SLE.

### Gut Microbiota and AD

The gut microbial diversity of AD patients has been found to be significantly different from non-AD population. The decreased microbial diversity observed in other conditions such as obesity, diabetes and Parkinson's disease was found to be linked to microbiome alterations (Keshavarzian et al., 2015). Furthermore, studies configure that these dysbiosis may be directly linked to neuronal changes principally through immune activation and inflammation (DuPont and DuPont, 2011). The alterations in the complex microbial population in the distal gut of AD patients may reflect direct changes in the gut-brain axis. One of the research studies has shown the role of gut microbiota in influencing the amyloid pathology (Harach et al., 2017). The mechanism configured was the presence of abundant gram-negative intestinal bacteria and LPS in people with AD, which ultimately led to the development of AD pathology.

LPS, an essential component of plasma membrane of gram-negative bacteria has been found to be associated with increased inflammatory response and increased mTOR activity. To validate this, a research study was conducted, in which peripheral blood mononuclear cell **(**PBMCs) were stimulated by LPS. It was observed that LPS induced phosphorylation of Akt, mTOR, and 4E-BP1, clearly indicating that LPS induced inflammatory response and this enhances the mTOR activity by enhanced phosphorylation. On inhibiting mTOR by rapamycin, it was seen that the levels of LPS induced IL-10, IL-6, and TNFα is greatly reduced, suggesting that mTOR regulation can effectively help in combating LPS induced inflammatory response (Schaeffer et al., 2012).

## Cancer and AD: a Common Biological Mechanism With Inverse Effects

“Amyloid hypothesis,” the production and deposition of fibrillar forms of amyloid-β and “tau hypothesis,” the abnormally phosphorylated tau with the formation of PHF's and NFT's are the two major causes leading to AD. Apart from amyloid and tau hypothesis, apoptosis and neuronal dysfunction also promote the advancement of AD (Green and LaFerla, 2008). In case of DNA damage, DNA repair pathways may be activated (but this reparative process is inoperative in cancer and AD. In cancer, it leads to unrestricted cell growth whereas in AD it leads to neuronal loss (Behrens et al., 2009). In context to carcinogenesis, mutations that inactivate p53, also activate mTOR particularly mTORC1, that promotes cancer by reverse glycolysis/Warburg effect and by promoting autophagy. In a hypothesis, Demetrius and Simon (2013) showed that metabolic deregulation plays a major role in cancer and AD. Cancer is distinguished by the overexpression of PFKFB3 (6-Phosphofructo-2-Kinase/Fructose-2,6 Bisphosphotase 3) enzyme which will enhance the glycolytic activity–a Warburg effect, whereas in AD, the expression of PFKFB3 in astrocyte, diminishes with age, and as a result, the process of the oxidative phosphorylation is upregulated–an inverse Warburg effect, to compensate the metabolic energy demands. Hence, the up-regulation of oxidative phosphorylation will lead to mitochondrial impairment and results in oxidative stress and ultimately in neuronal death. This metabolic perturbation of oxidative stress leads to the toxicity of proteins and lipids causing deposition of β-amyloid in the extracellular space of neuronal cells, a distinctive feature of AD (Demetrius and Simon, 2013).

### Skin Cancer and AD

There are several pathologic changes related to skin that have been found in patients with AD. A common enzyme present in both the conditions i.e., in AD and skin cancer is presenilin1 (PS-1). PS-1 acts a cofactor for γ-secretase cleavage of APP in AD (Xia et al., 2001). PS-1 is also involved in the catalytic cleavage of type I integral membrane protein, Notch receptor, required for cell fate, and neural differentiation events. It is evidenced that Notch signaling acts as a tumor suppressor in skin in the presence of p53 but dysregulation of Notch signaling may promote skin cancer. In other hypothesis, the increased expression of PS-1 leads to the accumulation of Aβ and tau, altering the cutaneous vascular function. Therefore, compounds that target the γ-secretase might also affect the PS-1 activity, affecting Notch signaling pathway, and this combined affect will lower down the Aβ production but will increase the probability of inducing cancer (Shih and Wang, 2007). Thus, care must be taken to make specific modulator which will only affect γ- secretase component without lowering levels of Notch intracellular domain (NICD) in the nucleus. Several studies have documented the role of mTOR in skin cancer pathogenesis. Overexpression of Akt in mouse models has shown increased probability of skin cancer on chemical induction. Thus rapamycin and rapalogs are being considered as a potential treatment in preventing skin cancer progression (Segrelles et al., 2007).

### Mitochondrial Dysfunction

Mitochondrion, a cellular organelle is engaged in diverse metabolic processes in the body including metabolism, calcium homeostasis, and apoptosis (Lim et al., 2007). Neurodegenerative disorders and various other diseases have been found to be linked with mitochondrial dysfunction, since mitochondrion performs activities such as energy production, cell growth, apoptosis, etc. (Chen H. et al., 2012). A research study on ASD (autism spectrum disorders) mouse model revealed that inhibition of mTOR by rapamycin has shown positive effects in terms of recovery from ASD. mTOR is known to get localized to outer mitochondrial membrane by binding of FKBP3 to FRB domain of mTOR where it anchors Bcl2 and Bcl-xl, involved in cellular apoptosis. Pavlov et al. have documented that APP processing by γ- and β-secretase produces Aβ in mitochondria, contributing to mitochondrial dysfunction (Pavlov et al., 2011). Hyperphosphorylated tau and NFT's, promote mitochondrial impairment which further obstructs energy production, enhancement of oxidative stress at the synapse, resulting in neurodegeneration (Ebneth et al., 2009). Aβ in itself has been seen to enhance tau pathology in transgenic mouse (Gotz et al., 2001). Evidences indicate that hyperphosphorylated tau disrupt mitochondrial function by obstructing mitochondrial transport, mitochondrial dynamics and mitochondrial bioenergetics (Wang et al., 2008). Apart from individual role, both Aβ, and tau also act in accordance with each other to directly affect the normal functioning of mitochondria. Specifically, the mitochondrial impairment integrates the close interplay of the two common hallmarks of AD, plaques and NFT's, or Aβ and tau, which act independently as well as synergistically on this vital organelle. Thus, strategies aiming to prevent mitochondrial dysfunction should focus on modulating or removal of both Aβ and tau pathology.

## Therapeutic Approaches for AD

### Rechaperoning

Both molecular (heat shock protein) as well as amateur chaperones [apo-lipoproteins (Apo-E)] and HS proteoglycans (HSPGs) play an essential role in chaperoning the misfolded proteins such as Aβ precursor protein (AβPP), perturb β-secretase (BACE) activity, inhibiting Aβ aggregation, and promoting internalization of Aβ into cells. Thus, HSPG's function as a dual role in the toxicity of Aβ and facilitates the clearance of Aβ in association with surface molecules in extracellular matrix (ECM). Modulating the HS-Aβ interaction with enzyme like heparanase could be a good therapeutic target for neurodegenerative diseases (Zhang et al., 2014). Moreover, it has been observed that Aβ-binding proteins and Aβ transporter proteins helps in clearing of Aβ across the BBB. Thus, by hindering Aβ from aggregating, Aβ-binding proteins like gelsolin, MG1 (a ganglioside) serves as a good therapy (Matsuoka et al., 2013).

**Figure 2:**
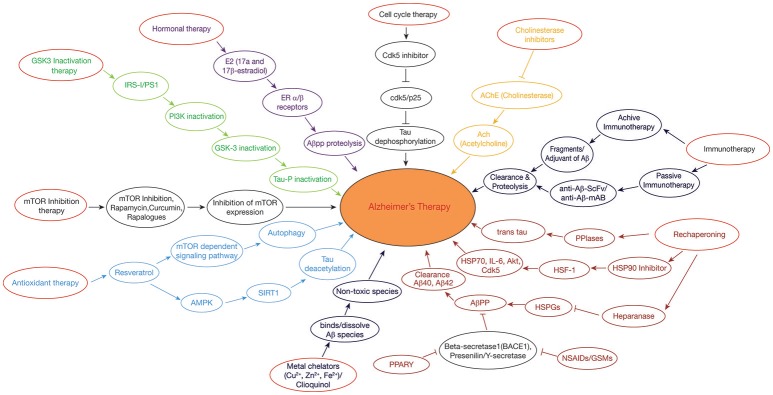
Therapeutic approaches in Alzheimer's disease: GSK3 Inactivation therapy: Neuroprotective compounds such as curcumin modulates PI3K/Akt/GSK3β pathway, leading to inactivation of tau hyperphosphorylation. Rechaperoning therapy: Modulation of HSPGs-Aβ interaction by heparanase; PPIases (Peptidyl-prolylcis/trans isomerases (PPIases) catalyzes the tau and APP from *cis* to *trans* forms, perturbing the amylodogenesis of Aβ. Antioxidant and Deacetylation Therapy: Resveratrol activates AMPK pathway which is in turn activates SIRT1, preventing tau from hyperphosphorylation by deacetylation. Hormonal therapy: E2(17β-estradiol) modulates the APP processing by activation of estrogen receptor β; Non-steroidal anti-inflammatory drugs (NSAIDs)/γ-secretase modulators (GSMs) and peroxisome proliferator-activated receptor-gamma (PPARγ) agonists regulate β-secretase by triggering APP through activated immune response. Metal chelating agents (Cloquinol, Cu, Zn, and Fe) are used to prevent the Aβ aggregation and amyloid formation. Cell cycle therapy: Cdk5 inhibitor functions as a neuroprotectant by preventing the proteolysis of p35–p25 pathogenic form. Cholinesterase inhibitor drugs maintain the level of acetylcholine in neuronal cells by inhibiting the enzymes from breakdown of the acetylcholine. Immunotherapy: Active and passive immunization is achieved against AD by using Aβ-42 with an adjuvant and anti-Aβ-42 (Sc-Fv&mAB), respectively. mTOR inhibitors: Rapamycin/rapalogs inhibit mTOR activity which in turn inhibits AD progression by suppressing hyperphosphorylation of tau and by promoting autophagy.

Peptidyl-prolyl cis/trans isomerases (PPIases) are typical class of protein chaperones and are very important for tau and APP biology. Tau and APP are proline rich intrinsically disordered proteins and are more sensitive to hyperphosphorlyation resulting in the formation of PHF's and NFT's. The PPIases perturb the oligomerization of tau and APP by catalyzing the tau and APP into *trans-forms*, as the *trans-*form is being more protective as compared to *cis* (Figure 2). PPIases consists of two main categories of proteins: the Parvulins and the Immunophilins. Furthermore, Cyclophilins (Cyps) and FK506-binding proteins (FKBPs) belong to the category of Immunophilins family. Yet some of the PPIases such as Pin1 and binding protein (FKBP) 12 are down regulated, whereas FKBP51 PPIases are up-regulated in case of AD. It has been shown that FKBP51 and FKBP52 along with HSP90 regulate the microtubule polymerization by coupling with tau. On the other hand, low molecular weight proteins like Pin1 belongs to parvulin family and (FKBP)12 catalyze the *cis* to *trans* isomerization of APP causing slow progression of amyloid peptide production (Cao and Konsolaki, 2011). Thus, to avoid the toxicity of Aβ plaques in AD, a balanced concentration of PPIases needs to be maintained. It is also seen that the oxidative stress can also be a cause for the oxidation of PPIase and thus the total PPIase concentration is lower in neuron and this contributes to the hyperphosphorylation of tau by regulating the activities of GSK3β and PP2A, resulting in the pathogenesis of AD (Blair et al., 2015).

### Immunotherapeutic Approach

Immunotherapeutic approach to treat AD takes into account the production of antibodies against Aβ, both actively as well as passively (Figure 2). In active immunization, low dose of Aβ is injected into a person along with an adjuvant such as polysorbate 80 and saponin to enhance the immunogenicity, (Figure 2) (Geylis et al., 2005). The drawback of active immunization lies in the hyperactivation of cytokines which may lead to neuroinflammation and autoimmune response. The first vaccine to be developed was (AN1792) which is made up of complete Aβ (1–42) peptide, synthesized *in vivo* along with QS-21 as an adjuvant (Schenk et al., 1999). However, its use was stopped as it posed the problem of T-cell mediated autoimmune reactions. Improved vaccines such as CAD106 and ACC001 have been prepared containing only B-cell epitopes to avoid T-cell posed autoimmune reactions (Ryan and Grundman, 2009). Several other vaccines are in the pipeline like MER5101 and AF205, presumed to offer best results with least side effects (Muhs et al., 2007).

While passive immunization demands the direct introduction of anti-Aβ antibodies which interact with the formed Aβ plaques and effectively work in their clearance. Passive immunization offers the advantage of reducing the toxic effects imposed by cytokines during active immunization (Figure 2). A humanized monoclonal antibody, Bapineuzumab (AAB-001), has been synthesized with an aim to facilitate clearance of Aβ clumps (Folch et al., 2018). Some other synthesized anti-Aβ antibodies include Solanezumab and Gantenerumab, in which, Solanezumab binds to soluble Aβ and Gantenerum Aβ binds to fibrillar forms of Aβ (Adolfsson et al., 2012). One limitation with passive immunization is that the synthesized monoclonal antibody is unable to cross the BBB due to its large molecular weight. Hence, this problem is now being overcome by designing single chain antibodies [single chain variable fragment (scFv)], which are being engineered in such a way that it has Fab region of both the heavy and light chains, known as fusion protein but without Fc region (Figure 2). This modification reduces its molecular weight due to which its delivery becomes trouble free. Two examples of anti-Aβ42 scFv antibodies include (Aβ1-16; scFv9) or (Aβx-42; scFv42.2) which is a target for N-terminal and C-terminal of Aβ42, respectively, thus helping in the clearance of Aβ plaques from AD brains (Huang et al., 2013).

### Hormonal Therapy: Estrogens in AD

Estrogens are being considered as potential drugs for the prevention of AD and its associated neuropathological changes such as the blocking of Aβ formation, tau hyperphosphorylation, NFT's formation, oxidative damage, and neuro-inflammation. Estrogens, like estrone (E1), estradiol (E2 or 17β-estradiol), and estriol (E3) are bestowed with the power of reducing Aβ production and fibrillation (Figure 2) (Behl et al., 1997). Researchers have shown that activation of estrogen receptor β prevents the toxicity of Aβ protein through the signaling pathways of PI3K/Akt and MAPK/ERK (Si et al., 2012).

Scientific data has put forward that estrogens offer neuroprotection by maintaining constant mitochondrial membrane potential against the actions of mitochondrial toxins such as MPTP (mitochondrial permeability transition pore) (Kenchappa et al., 2004) and 3-nitroproprionic acid (3-NPA) by maintaining calcium homeostasis (Nilsen and Brinton, 2004) and oxidative stress in mitochondria.

### PPARγ Agonists as Therapeutic Approach in AD

The peroxisome proliferator activated receptor (PPAR) is a representative of nuclear receptor family that participates in the adipogenesis (Issemann and Green, 1990). Along with this, PPARs (ligand-inducible transcription factors) participates in diversified functions including cellular differentiation, metabolic regulation, and homoeostasis (Bogacka et al., 2005). PPARα (NR1C1), PPARβ/δ (NR1C2), and PPARγ (NR1C3) are the three members of the PPAR family. An abnormality in any of the member of PPAR family severely affects normal functioning of CNS, indicating the pivotal role played by PPAR in sustaining the usual functioning of the nervous system. Experimental data suggest that treating AD with PPARγ agonists such as Pioglitazone, Ibuprofen, significantly decreases the levels of the proinflammatory enzymes Cyclooxygenase (COX-2) and induces Nitric oxide synthase (iNOS), thus reducing amyloid plaques in the neuronal cells. Also, PPARγ over expression has been found to prevent H_2_O_2_-induced ROS (reactive oxygen species), stabilizing mitochondria and ultimately lowering Aβ-induced toxicity (Fuenzalida et al., 2007). Researchers have shown that Non-steroidal Anti-Inflammatory Drugs (NSAIDs) serve as PPARγ agonists which perturb the progression of Aβ amyloid formation by modulating the action of inflammatory cytokine response by blocking the expression of interleukins such as IL-6, IL-1β, and COX-2 or inhibits the expression of BACE-1 which interferes in the catalysis of amyloidogenic APP (Figure 2) (Combs et al., 2000).

Furthermore, Thiazolidinediones and its derivatives obstruct AD progression either by inducing Aβ for ubiquitination pathway, also known as Aβ proteolysis or by enabling the β-secretase for inhibition. In addition, activated PPARγ also works in hindering AD pathology by inducing Aβ endocytosis and down regulating the levels of cytokines secretion through the modification of inflammatory response (Mandal et al., 2006).

### Resveratrol, a Neuroprotective Compound

Diversified data exhibits that resveratrol containing compounds play a neuroprotective role (Karuppagounder et al., 2009). For example, red wine rich, resveratrol (3, 4, 5-trihydroxy-trans-stilbene), is a polyphenol compound, which is found to be indulged in diversified pathways such as anti-inflammation, antioxidant, and autophagy promotion which ultimately aims at abstaining AD (Orgogozo et al., 1997). Evidences have shown that resveratrol modulates autophagy and other pathological symptoms of AD by activating AMPK, SIRT1, and PPARγ receptor (Um et al., 2010).

Resveratrol activates AMPK, by phosphorylation at Thr172 through the action of Ca^2+^/CaM-dependent protein kinase (CaMKK). Activation of AMPK inhibits mTOR activity which further enhances autophagy ultimately resulting in Aβ clearance (Figure 2), (Hawley et al., 2010). Resveratrol also acts as an activator of SIRT1 which deacetylase tau and this promotes Aβ autophagy (Figure 2) (Borra et al., 2005). Resveratrol protects mitochondrial functions and works in eliminating free radical species and repressing microglia activation (Yang et al., 2016). Thus, resveratrol acts as an anti-inflammatory agent and as an antioxidant by modulating miRNAs and therefore it functions as a neuroprotective agent.

### γ-Secretase Inhibitors (GSI) and Modulators (GSM)

Aβ is produced when APP is processed by a protease known as γ-secretase; the process is termed as amyloid hypothesis. Thus, γ-secretase is becoming an area of interest for researchers to obstruct Aβ production. The mechanism behind the production of Aβ is the catalytic cleavage of APP by both β- and γ-secretase. APP is first cleaved by β-secretase which generates a 99 residue fragments from APP and is further cleaved by γ-secretase and ultimately, producing Aβ. Among various Aβ variants, Aβ42 is a distinguished feature of AD. Multiple inhibitors of γ-secretase have been developed such as Difluoroketonepeptidomimetic which is known to inhibit keto- or aspartyl-forms of both serine and cysteine protease which hinders the formation of PHF's and NFT's (De Strooper et al., 2012). Hydroxyethylamines and Hydroxyethylureas are another genre of GSI's which are known to block aspartylprotease by representing as a transition state analog (Li et al., 2000). Although these, GSI's have offered remarkable way to combat Aβ production but the major problem concerning GSI's is that apart from inhibiting γ-secretase, it also interfere the catalytic breakdown of Notch protein in the membrane which disrupt the signaling cascades leading to a disease (Zhang et al., 2013) (Figure 2).

To overcome this problem, scientist community is focusing on developing γ-secretase modulators (GSM's) rather than GSI's. GSM's work by replacing the cleavage site of γ-secretase on APP due to which more number of other Aβ variants is produced and production of Aβ42 is reduced considerably. Reduction in Aβ42 helps in solving the problem of Aβ aggregation and ultimately the formation of PHF's and NFT's is reduced. GSM's in use today are of three categories, i.e., NSAID-derived carboxylic acid GSMs, non-NSAID heterocyclic GSMs, and natural product derived GSMs. These GSM's modulate the activity of γ-secretase in such a way that, its cleavage action on APP lowers Aβ42 levels while upregulates the levels of small peptide fragments (Aβ37 and Aβ38) which are comparatively less toxic in nature (Xia et al., 2012).

### Metal Chelating Agents

Miscellaneous transition metal ions such as copper, zinc, iron play a principal role in various metabolic processes of the cell. These metal ions specifically copper ions are found to be present in Aβ clumps, holding them together, whereas iron and zinc have the power to bind hyperphoshorylated tau forming NFT's (Kozlowski et al., 2009). The existence of metal ions in the neuronal cells becomes even more crucial as these metal ions are susceptible to form ROS by undergoing redox reactions. Therefore, stable balance of these metals needs to be maintained for proper functioning. Interference in the metal ion homeostasis or a hindrance in the connection of Aβ with these metal ions has been found to pose a number of neuropathological changes, also known as metal hypothesis (Gaeta and Hider, 2005). In view of this, major focus is on to disrupt the connection between Aβ and metal ions by chelating these ions using metal chelators so that Aβ amyloid will not be able to aggregate (Figure 2). Desferrioxamine B was the first to be used for this purpose; however, its further use has been stopped due to its various side effects as it tightly binds to Fe (III) which almost made this ions concentration negligible, leading to anemia (Zatta et al., 2009).

Keeping this in view, Clioquinol (5-chloro-7-iodo-8-hydroxyquinoline, CQ), a small lipophilic compound has been made which can easily cross the BBB and functions not by binding to metal ions but by removing them from Aβ clumps, in such a way that dissolves the Aβ aggregates, thus resisting AD progression (Cherny et al., 2001).

## Restricted Cell Cycle Re-Entry as Therapeutic Approach for AD

Research study has proved that like cancer, AD is also a result of dysfunctional cell cycle. Evidences indicate that abnormal cell cycle results in one of the two possibilities; firstly, neuronal cells may enter into apoptosis. Secondly, it may trigger chronic oxidative damage which prevents apoptosis, but gaining “immortality” similar to tumor cells (Moh et al., 2011). In any of the case, amyloid plaques, NFT's and Aβ aggregates are formed throughout the brain tissue. Thus, it is proposed that cell cycle inhibitors can effectively work against AD.

Neuronal cells do not follow normal cell cycle cascades as like other cells rather they remain in the G0 phase of the cell cycle as they are non-replicating, but under certain conditions these neuronal cells exit G0 phase and are forced to re-enter the cell cycle (Lopes et al., 2009). It is well known that for the creation of synapses, neuronal cells have to continuously polymerize microtubules which results in phosphorylation of tau, leading to NFT's (Ogawa et al., 2003). Apart from the production of hyperphosphorylated tau and NFT's, this forced re-entry also poses the problem of oxidative damage in neurons. The forced re-entry of aberrant neurons into cell cycle phase, results in their abortosis instead of apoptosis. The oxidatively damaged neurons in AD brains secrete α- and γ-secretase to produce Aβ by cleaving APP, as Aβ is thought to be an antioxidant, neutralizing the free radicals that are generated. However, over production of Aβ also act as a mitogenic factor for the forced re-entry of neurons into the cell cycle. It is also evidenced that accumulation of Aβ in neuronal cells activates a protease, Calpain, which ultimately cleaves the Cdk5-p35 bond and forms a more stable bond of Cdk5/p25 (Shah and Rossie, 2018). Hyperactive response of Cdk5/p25 has been seen to phosphorylate various proteins such as tau which affects microtubule stability leading to the formation of PHF's and NFT's, which ultimately contribute to AD (Figure 2).

Furthermore, fluctuations in the concentration of Cyclins and CDKs, also promote the re-entry of neurons into the cell cycle cycle through G0-G1 phase and beyond. Research study has given an indication that Cyclin D, Cdk4, Cdk5, mitotic signaling G-protein Ras, PCNA (Proliferating cell nuclear antigen), a S phase marker, have been found to be up-regulated in neuronal cells of AD brains, due to which these cells exit the G0 phase and re-enter the cell cycle (Zhu et al., 2004). The ubiquitination process is also transformed in AD due to which proteins destined for degradation could not be degraded, consequently giving rise to accumulation of misfolded protein, eventually gaining immortality by escaping the normal apoptotic pathway (Haapasalo et al., 2010). Thus, blocking the re-entry of neuronal cells into the cell cycle can help in preventing AD progression. Epigallocatechin-gallate, Tamoxifen, Retinoic acid, Rosiglitazone are some of the cell cycle inhibitors which serve this purpose (Figure 2) (Wu et al., 2010).

## Conclusion and Perspective

Overexpressed mTOR, known to be the probable cause of AD, is found to be regulated with a number of upstream signaling cascades such as (PI3-K)/Akt, GSK3, AMPK, IGF-1. It has been seen that various diseases such as cancer, autoimmunity, mitochondrial dysfunction etc. deregulate these pathways which hyperactivate mTOR leading to hyperphosphorylation of tau, forming PHF's and NFT's, a hallmark and an initiator of AD. mTOR also contributes to the production and aggregation of Aβ plaques by directly inhibiting autophagy. Accumulated Aβ induces tau hyperphosphorylation and mTOR activation, which further enhances AD progression. Consistent with these predictions, the inter-relationship between mTOR-tau-Aβ has been viewed as an attractive avenue to propose possible therapeutic approaches. The most important aspect is to develop therapies which aim at maintaining homeostasis, i.e., inhibiting AD development without perturbing normal signaling pathways. In this regard, structural/functional modulators of γ-secretase rather than cell cycle and γ-secretase inhibitors would be an appropriate therapeutic approach. Moreover, research study should focus on developing compounds that target the disrupted signaling pathways with minimal side effects such as blocking the hyperphosphorylation of tau without affecting its normal physiological action.

Despite applying multifarious approaches, there is still a huge gap between AD pathogenesis and its successful treatment. This is mainly due to incomplete understanding of the role of several components in AD pathogenesis such as mTOR, tau, Aβ, aging, oxidative stress, autophagy, etc. Moreover, the cross interplays among these components and the mutual relationships in their mechanism to promote AD are still a question of debate. Over two decades of research has been focused on how amyloid pathology is one of the main culprits for AD development. However, contrary to this, continued research led scientists to proclaim that improved animal modeling suggests a key role for Aβ dyshomeostasis in initiating AD (Selkoe and Hardy, 2016). In contrast to this, Kametani and Hasegawa, have given a clear indication of the fact that tau and not Aβ is the main underlying cause behind AD pathogenesis. Their findings are based on the fact that various experimental mouse models with increased Aβ deposits in brain did not show any NFT's formation or neurodegeneration. Moreover, they showed that several AD patients with few Aβ deposits and normal people with a great Aβ deposits have been observed which further confirm that Aβ is not toxic to neuronal cells (Kametani and Hasegawa, 2018). Recent finding of 2018 suggest that dysfunctional liver metabolism may also be a contributing factor in Aβ depostis. However, results are intruiging which demands further study research to clearly specify the role of liver in Aβ degradation (Maarouf et al., 2018). It is known that aging is a major factor in AD and other neurodegenerative diseases and attenuating aging can prove to be a boon for treating AD but the question of concern is to develop techniques, strategies or drugs that all aim to prevent AD by delaying aging without hindering the normal functioning of mTOR (Galvan and Hart, 2016). Since mTOR deregulation has been found as a hotspot for cerebrovascular dysfunction which accounts for a cause behind AD progression. Therefore, mTOR dependent cerebrovascular protection may offer a suitable target to develop strategies to prevent AD progression or other age related diseases that share the common etiology of cerebrovascular dysfunction while avoiding undesirable side effects (Van Skike and Galvan, 2018).

## Author Contributions

ZM generated the concept of this manuscript. PT searched the literature and arranged the references. SM produced figures and reviewed the written matter. RD arranged the manuscript written by different authors in publishable format. MK critically reviewed the manuscript and suggested the prescribed journal. NP reviewed the whole manuscript and finalized it as per publisher's instructions and submitted the article. All the authors have formally agreed on the content and their credit of contribution.

### Conflict of Interest Statement

The authors declare that the research was conducted in the absence of any commercial or financial relationships that could be construed as a potential conflict of interest.
